# “Partners rather than just providers…”: A qualitative study on health care professionals’ views on implementation of multidisciplinary group meetings in the North West London Integrated Care Pilot

**DOI:** 10.5334/ijic.2019

**Published:** 2015-09-02

**Authors:** Angelos P. Kassianos, Agnieszka Ignatowicz, Geva Greenfield, Azeem Majeed, Josip Car, Yannis Pappas

**Affiliations:** Department of Primary Care and Public Health, Imperial College London, London, UK; Social Science and Systems in Health, Warwick Medical School, University of Warwick, Coventry, UK; Department of Primary Care and Public Health, Imperial College London, London, UK; Primary Care, Department of Primary Care and Public Health, Imperial College London, London, UK; Department of Primary Care and Public Health, Imperial College London, London, UK; Health Servicers Research, Institute of Health Research, University of Bedfordshire, Luton, UK

**Keywords:** integrated care, health services, health care professionals, qualitative, collaboration, multidisciplinary groups

## Abstract

**Introduction:**

Multidisciplinary group meetings are one of the key drivers of facilitating integrated care. Health care professionals attending such groups have a key role in the success of these discussions and hence, in the forming of multi-professional integrated care. The study aimed to explore the professionals’ experiences and views of participating and implementing the groups in integrated care context.

**Methods:**

A qualitative study including 25 semi-structured interviews with professionals participating in the Northwest London Integrated Care Pilot analysed using thematic content analysis.

**Results:**

Participants mentioned a number of benefits of participating in the meetings, including shared learning and shared decision-making between different services and specialties. Yet, they perceived barriers that diminish the efficiency of the groups, such as time constraints, group dynamics and technicalities. The participants felt that the quality of discussions and facilitation could be improved, as well as technical arrangements that would make them easier to participate. Most of the participants perceived the groups to be beneficial for providers mostly questioning the benefits for patient care.

**Conclusion:**

Findings provide an insight into how health professionals’ views of their participation to the multidisciplinary group meetings can be more effectively translated into more tangible benefits to the patients. To benefit patient care, the multidisciplinary groups need to be more patient-oriented rather than provider-oriented, while overcoming professional boundaries for participating.

## Introduction

### Health care challenges and integrated care

For years, the lack of integration has been a barrier for quality care for health services users. Recently, lack of integration was perceived by stakeholders as the greatest weakness of the health care system [1]. The objects of integration can be functional, organisational, professional or clinical [2]. Moreover, the range and level of integration can vary from “linkage” to creating new organisational infrastructures [3]. The need for better integration of care is a long-standing concern in the English and Welsh National Health System. Several European countries currently implement national plans to promote integrated care to cope with the challenge of care for people with long-term conditions [4]. Currently, the National Health System in England is driven into focusing on integrated care because of the increasing ageing population [5]; the increasing prevalence of multiple long-term conditions; the significant proportion of people in acute hospital care who have dementia as a comorbidity; several concerns raised about quality of care provided and the need to improve quality of care because of the economic crisis; and also the need to contribute to financial savings without compromising quality of care or efficiency [6] through more efficient ways of working [7]. However, recent evaluations of integrated care systems have provided mixed results on its efficiency [8–10], with evidence indicating that planned admissions and outpatient attendance were reduced but emergency admissions were not, whilst patient experience was not improved [11].

### Integrated Care Pilot in North West London

In July 2011, National Health System North West London established a programme of health and social care integration known as The North West London Integrated Care Pilot. The pilot was a large-scale programme focusing on developing new models of care planning and coordination for people with diabetes and those who are over 75 years of age [9]. It used a mixed interventions approach, involving a total 38,000 patients (8700 over the age of 75 diagnosed with diabetes, 6500 under the age of 75 diagnosed with diabetes and 22,800 elderly patients without diabetes) from 103 general practices covering 550,000 patients, two hospitals, five Primary Care Trusts, three Community Health Trusts, two mental health providers, five social care providers and two non-governmental organisations [12]. The Integrated Care Pilot's objective was to reduce emergency admissions and provide better access to care outside the hospital for patients with diabetes and complex care needs. The organisations participated in the Integrated Care Pilot voluntarily and representatives were expected to work collaboratively and to share their expertise to reach the aims of the pilot. The organisational structure and processes of the Integrated Care Pilot are described in detail elsewhere [9, 12, 13].

The Integrated Care Pilot encompassed three main interventions, which aimed to provide better and integrated care: care planning, information sharing tools and multidisciplinary group meetings, as well as shared governance among health and social care organisations. Multidisciplinary group meetings were formed to coordinate the organisation and delivery of health care across the community, primary and secondary care services. All participating organisations were represented in the multidisciplinary group meetings. Health care professionals who represented their organisations were provided with objectives for integration as a vehicle for enhancing productivity and efficiency within and across organisations. The multidisciplinary group meetings convened to discuss patient cases and comprised of general practitioners and representatives from the acute, mental health and social care trusts. Among others, multidisciplinary group meeting participants included general practitioners, psychiatrists, diabetes specialists and mental health representatives. Multidisciplinary group meetings were seen as key facilitators of integration, as moving from individual to multidisciplinary decision-making, and from a focus on conditions to a focus on individualised care. They were also used to facilitate and deliver joined-up care [14].

There were 16 multidisciplinary group meetings convening once every month in North West London. The main purposes of the multidisciplinary group meetings were to consider care plans for complex cases of patients and to reduce the number of hospitalisations. During the meetings, the general practitioners in turns brought a complex patient case to the group for discussion around delivering coordinated care to the patient. Also, multidisciplinary group meetings were used as a forum for health professionals to exchange experiences and information related to a variety of health care services. Each multidisciplinary group meetings held registers for patients >75 years old who had diabetes and used the integrated care programme information sharing tools to stratify these patients based on their risk of emergency admission. Each multidisciplinary group meetings went through a mobilisation stage from formation and governance through care planning roll out.

### Multidisciplinary groups in health care

The multidisciplinary group meetings in the North West London Integrated Care Pilot constitute a forum for health professionals to understand the concept of integration and make necessary changes to their everyday practice [15]. Therefore, ideally health professionals should be able to transform shared knowledge into practice. Recent evaluations did not point this out [8–10]. There are multiple challenges when health care professionals are required to work together which include lack of standardised procedures [16]. In an organisational or systemic level, decision-makers are called to clarify the process, structure or outcomes of the multidisciplinary groups to help health professionals work together because health professionals are more comfortable working alone [17]. For example, in an integrated care context, complementary as opposed to competitive behaviour can benefit successful integration [18]. Therefore, complementary behaviour together with enhanced understanding of the standardised procedures can lead to better outcomes. Consequently, multidisciplinary groups have the potential to improve communication and improve patients’ understanding of the care process [19]. It is promising that recent evidence indicates that young medical, nursing and other health professional students are positive towards inter-professional collaboration and learning [20].

Health and social care professionals have a crucial role in delivering integrated care and when participating in multidisciplinary group meetings. The views of professionals are important because they are responsible for delivering health care and constitute the facilitators of integrated care. The interaction between professionals with different background requires mutual respect, trust and collaboration [21] as well as quality of communication [22]. Professionals’ perceptions of multidisciplinary team working can provide a framework of knowledge to inform and improve future interventions to benefit patient experience and reduce health care costs. National Health System of England provides a range of opportunities to explore the outcomes of multidisciplinary team working and its effect on quality of care [23]. The outcomes of multidisciplinary group meetings can be associated with organisational, systemic and interactional aspects of health care [21]. Multidisciplinary group meetings can provide a platform for shared decision-making and recent findings point out that professionals in the Netherlands consider lacking tools to facilitate shared decision-making as problematic [24]. Moreover, a recent study on stakeholder's perceptions of health care reforms in Belgium highlighted that lack of incentives like attending multidisciplinary group meetings constitute a barrier for integration of care [1].

Professionals’ management, leadership and shared values define their participation in and influence on the multidisciplinary group meetings [9]. Specifically, most talk in the multidisciplinary group meetings in the Integrated Care Pilot was exchanged between medical professional [25]. Also, one way to facilitate equal participation between different professionals [13] is to understand what multidisciplinary teams do and how they do it [26].

The multidisciplinary group participation and collaboration was found to benefit patient health care in general [27–29] as well as clinical outcomes (i.e. hospital admissions) and patient experience (i.e. quality of life) [30]. Previous studies on professionals’ views on multidisciplinary group meetings pointed out the inadequate funding from the health system [1] and their shared view that they use them to benefit themselves by providing opportunities for improving education, communication and efficiency as well as fostering professional relationships [31]. However, this was limited to multidisciplinary cancer care. To our knowledge, there are no other studies qualitatively exploring professionals’ views on multidisciplinary group collaboration in the context of integrated care. This study, aimed to explore the experience of forming integrated care through multidisciplinary group meetings from professionals’ perspective in the context of the Integrated Care Pilot in North West London.

## Methods

The findings reported in this paper form part of a mixed-method evaluation that explored perceptions, experiences and involvement of providers and users in the North West London Integrated Care Pilot, UK [9, 10, 14, 32].

### Sampling and recruitment

Between autumn 2011 and spring 2012, 16 semi-structured interviews were conducted with professionals delivering care and implementing the Integrated Care Pilot. A purposive sampling strategy was adopted to maximise the representativeness and diversity of professionals involved in the initiative.

We contacted over 75 professionals via email and/or telephone and invited them to take part in the interview. The 16 providers we interviewed included 8 general practitioners, 2 geriatric specialists, 1 end of life facilitator, 2 psychiatry consultants, 1 diabetes specialist, 1 social worker and 1 practice manager. The study received ethics approval by the National Health System National Research Ethics Service for City and East London (ref 11/LO/1918). Informed Consent was obtained from participants before conducting the interviews.

### Interviews

The interview schedule was designed to support the development of an understanding of structure and process of the Integrated Care Pilot with a view to exploring the ways in which the multidisciplinary group meetings promote integration of care and, ultimately, efficiency and improved patient outcomes. The North West London Integrated Care Pilot did not set out to find only about multidisciplinary group meetings but the multidisciplinary group meetings were only a small component of all the integrating processes. Therefore, participants were not asked very detailed questions about the multidisciplinary group meetings but in the end, all participants talked about the multidisciplinary group meetings. The process of thematisation of questions and probes can be found in the Study Protocol (see Appendix).

All interviews were conducted face-to-face at the participants’ workplace or a chosen location for convenience. The interviews lasted between 25 and 45 minutes and were audio-recorded and transcribed verbatim by a professional company providing medical transcription services.

### Data analysis

All interviews were checked for accuracy and participants’ and their organisations’ names were anonymised. Analysis of data was thematic, using a constant comparison [33]. Three researchers conducted the data analysis and coding independently and codes were then discussed within the research group of experienced qualitative researchers. Codes were created both horizontally (by coding each interview as a standalone hermeneutic unit) and vertically (by scanning across the data for specific terms) and then developed into categories and themes. Categories were refined and coding reviewed throughout the process for which the Atlas^®^ software [34] was used. Thematic saturation was reached after the first 16 interviews as no new or relevant information emerged [35]. Also, no other sub-themes were explored because of the focused research question of the study which was health professionals’ experience of participating in the multidisciplinary group meetings in an integrated care context. Moreover, this study forms part of a broader evaluation, and the questions that were explored around the multidisciplinary group meetings were only a small subset of all questions in the interview schedules (see Appendix).

## Results

**Figure 1.**
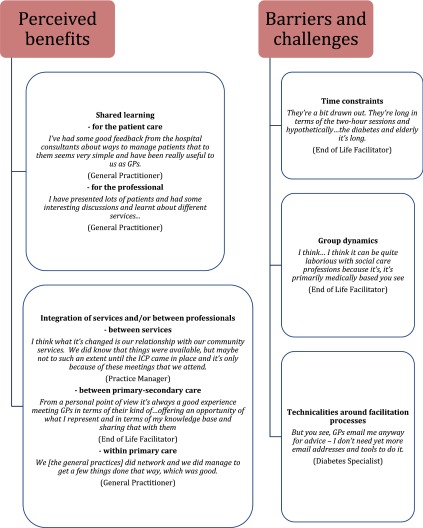
Examples of health professionals’ narratives of benefits and barriers of participating in the multidisciplinary group meetings.

Two themes were identified related to the professionals’ perceived benefits and barriers/challenges for participating in the multidisciplinary group meetings. A third theme was the result of evaluating these benefits and barriers by providing suggestions for overcoming barriers. Perceived benefits were explored around the shared learning processes and technicalities around the facilitation processes. The barriers were mainly organisational. Figure 1 provides representational quotes that inform the benefits and barriers from our analysis. No interesting conclusions were drawn regarding any differences between the meetings. Moreover no differences between patient populations were addressed as the North West London Integrated Care Pilot was targeting health professionals working with patients with diabetes only. However, some interesting differences were found between general practitioners and other health professionals, and these issues are described.

### Perceived benefits of multidisciplinary group meetings

Participants mentioned several benefits from attending the multidisciplinary group meetings. They expressed these in terms of learning and sharing information between different services and specialties. All of the perceived benefits focused around the multidisciplinary nature of the multidisciplinary group meetings. Professionals accounted for learning in terms of sharing information, joint accountability and incentives. The multidisciplinary group meetings were seen as a vehicle to share knowledge with other specialists. Even though there was a general agreement amongst most of the primary care practitioners that the benefits of multidisciplinary group meetings extended to both the professionals and the patients this was not see in all health professionals who reflected in mostly benefits for themselves rather than patient care.

#### Shared learning

Professionals perceived shared learning in the context of multidisciplinary group meetings as beneficial. However there were doubts if the learning translated to patient benefits. One participant points out that the professionals may “have presented lots of patients and had some interesting discussions and learnt about different services, but it is rare for there to be a major impact on patient care”.

The benefits of shared learning can also be used as a construct to understand other perceived benefits outlined below like integration between services and/or professionals. The learning benefits for most of professionals were reflected within the context of their clinical practice. One primary care specialist spoke about the impact the multidisciplinary group meetings had on managing patients in the context of learning “…a lot, and I'm sure everyone else is, because we do, we learn as we go, no matter how experienced you are” (General Practitioner 1).

Another primary care professional elaborated on the long-term benefits of shared learning:

“What I learnt, it's not about actual how to manage this patient now, it's about learning how learn for other patients, like, in this sort of situation, this is the sort of thing you would do” (General Practitioner 5).

The multidisciplinary nature of the meetings provided the opportunity for primary care specialists to develop their knowledge and improve the understanding and awareness of self and others within the local health care economy. For some, it also helped to foster a change in how they managed patients:

“It's changed my management in a way, for example I just visited a patient where I'm now actually thinking rather than just her blood pressure and her cholesterol and different things, I'm now going what's her continence like? What's here social care like? What's her memory? What's her mood? In my mind I've far more of the memory clinics, the district nurses; it's sharpened my mind very much to what's there, a more holistic approach sometimes” (General Practitioner 6).

A secondary care specialist reflected on the fact that primary care specialists surprisingly valued their input.

“I think I've been pleasantly surprised how my input has been valued. I didn't expect it to be as valuable as it appears to have been to some of the general practitioners. And certainly, anecdotally, they've said they've enjoyed the meetings. They've enjoyed the learning. But one of the things they did enjoy perhaps more than bringing their own cases was when I brought cases from the hospital. So that was quite helpful for them, because, you know, they were seeing the kind of problems we're having to deal with…” (Geriatric Specialist).

#### Integration between services and between professionals

Integration benefits were perceived by professionals in three levels: (a) bringing together people from different services allowing collaboration (integration of services), (b) bringing together people with diverse backgrounds (integration between professionals) and bringing together primary care specialists from different practices (integration within primary care). Primary care specialists viewed this as an opportunity to network and learn more about available community services. Integration of services was also seen as a mean of enhancing knowledge and understanding of specific conditions.

Integration of care between primary and secondary care specialists was considered as beneficial in terms of exchanging experiences. These benefits of sharing knowledge and bridging the gap between primary and secondary care was perceived as fostering relationships.

“I think one of the areas that I've been most in favour of this, has been the closer integration with Primary Care and actually meeting up with local GPs. And this in a sense is bringing them closer together again, where they get to learn from us and we get to learn from them and so it's an exchange…” (Geriatric Specialist).

On the other hand, fostering relationships were not restricted between primary and secondary specialists. Multidisciplinary group meetings were also seen as a vehicle of bridging the gap between general practices in terms of establishing contacts between neighbouring practices.

“The MDGs are the best part of it [the ICP] really. So, that is the one fundamental thing that I found useful. It's quite nice to meet your neighbouring practices and for them to bring along a clinical case, for us to bring along a clinical case, to have just as the name suggests, different people from different disciplines…” (General Practitioner 4).

In the integrated care context, patient discussions were viewed as potential opportunities for participants to examine the broader inefficiencies and challenges in the inter-organisational environment.

“What GPs need on the ground is the immediate and then the learning will follow. Give me the consultant to phone when I've got an elderly lady at home that I've got difficulty coping with…This is what we need. We don't want words and talk and meetings and so on. And this is what it's all about in the end. Its talk and words” (General Practitioner 9).

As the GP in the quote above reflected, in order to facilitate integration of services, a “joined-up” working which will affect the care of many patients not just the only the ones that are discussed in the meetings, a very different approach is required.

### Barriers and challenges for participating

Even though most of the participants accounted for a number of perceived benefits of their participation in the multidisciplinary group meetings, they also accounted for several barriers and challenges. These were mostly around time constraints, the group dynamics in discussing patient cases and several technicalities related to the meetings restricting participation.

#### Time constraints

The issue of time was reported by most of the participants as a barrier for physically attending the multidisciplinary group meetings. Participants pointed out that multidisciplinary group meetings were long and time consuming. For example, the frequency of weekly multidisciplinary group meetings restricted professionals developing other areas of work.

“And there have been a few MDG groups set up and it is quite difficult, because you end up, virtually every week you are spending an afternoon at one of these meetings which means that it's leaving less time for you to develop your other services” (Geriatric Specialist).

These time commitments were often rationalised by weighing the perceived benefits of the multidisciplinary group meetings. For instance, one professional's decision to attend was based on his judgment of learning to be acquired and the importance of his day-to-day commitments questioning whether “is that time well spent, compared to other things?” Participants also highlighted that even though they were reimbursed for their participation in the multidisciplinary group meetings, seeing their patients was also – if not equally – maybe more important.

“So, meetings twice a month and it's too much, really, and for two hours. And so that was too much to ask for. And then the usefulness of the meetings, because there was quite a wide range, social services and the consultants and the community services, which was nice… On the other hand, we weren't sure about how much learning there was” (General Practitioner 5).

#### Group dynamics

The facilitation process of the multidisciplinary group meetings was also briefly but importantly seen as a barrier to participation for those who believed the meetings were medically focused. This contradicts the idea that emerged in the previous participants’ comments where the collaboration between medically and non-medically trained providers was deemed as useful. Even though group dynamics are briefly mentioned as a barrier for participating in the multidisciplinary group meetings, they are needed in order to put health professionals’ multidisciplinary work in context and expand in how non-medically trained health professionals may experience their contribution to the multidisciplinary group meetings.

“I think … I think it can be quite laborious with social care professions because it's, it's primarily medically based you see” (End of Life Facilitator).

Various participants observed variability between the meetings in terms of the quality of the discussions taking place, not only because of different facilitation approaches but also because of the attitudes and motivation of those participating. These restrictions may have reflected the underlying group dynamics within the multidisciplinary group meetings.

“The other issue is that if the variability from buying from different … so there's some MDGs that I go to the case conferences and everyone is quite academic and quite motivated and doesn't want to make a fool of themselves in front of other people so they bring good cases and have really sophisticated conversations and that doesn't happen everywhere” (Psychiatry Consultant 1).

#### Technicalities around facilitation processes

Several participants elaborated on how the content of the multidisciplinary group meetings was organised. The content in some cases lacked the depth of information provided.

“Because some of the presentations that they gave were, historic to some extent or felt like actually, you know, the information they were presenting, even … particularly from a social care perspective was very … it was quite, you know, it wasn't thought about in terms of what they would elicit information” (End of Life Facilitator).

Another participant suggested that the quality of the discussion within the multidisciplinary group meetings was “bad” with no potential benefit for participants or patients.

“I've got to spend Saturday morning doing my matters at [a hospital] because I spent four hours in an MDG that was … and you know, the ten GPs there only because they were being paid, the quality of what was discussed was, you know, bad … For what?” (Diabetes Specialist).

One of the participants provided a rationale for lack of interest to multidisciplinary group meetings by discussing the preference towards the old network meetings where the focus was on collaborative work (“working together”) rather than discussing individual cases.

“Well, what people say is that some of the multidisciplinary group meetings that work well, you know, work well – you know, people quite like coming together. But you see, we used to have network meetings … we used to discuss more working together, rather than individual cases” (Diabetes Specialist).

One of the things that participants elaborated on was how the cases were organised and brought to the multidisciplinary group meetings. Some of the participants perceived the multidisciplinary group meetings as a forum for discussing the “difficult” cases but as the pilot progressed and as a result of a lack of clear guidelines, other patient cases were also discussed. Amongst the comments about the multidisciplinary group meetings, was short notice to attend the meetings, lack of organisation when it comes to having different multidisciplinary group meetings collapsing at the same time and the lack of outcomes coming from the multidisciplinary group meetings with several considerations not taken into account.

“So I think at the beginning what we were seeing in the multi-disciplinary meetings, the MDG meetings, where GPs were bringing patients who they know were difficult. So all the hard cases were brought up to begin with and then as the pilot went on they were, sort of, struggling to know what to bring. So one of the things we suggested was just to bring two or three cases from their highest risk bracket” (Geriatric Specialist).

### Overcoming barriers for participating

As a result of barriers and challenges reported, a third distinct theme was related to participants’ evaluations of the need, usability and feasibility of the multidisciplinary group meetings providing suggestions for overcoming barriers for participating. Their comments focused on the implementation processes both at the organisational and individual level. When referring to implementation processes we refer to the discussion between participants within the multidisciplinary group meetings, organisational issues and the multidisciplinary group meeting outcomes including learning outcomes.

“As a secondary care physician, it was really nice to get out of the hospital, it was really nice to go and meet GPs and put faces to names. It's really nice to have in depth clinical conversations about complex patients and work together in a creative way to come up with ideas. I think the case conferences for this are the best model … and here we've got a system that's trying to help us; it makes it easier. It's really rewarding clinically. The frustrating aspects of it, we've just had a long conversation about how to try and improve this when people don't bring cases or people don't … you spend a lot of time thinking about the case and then actually they don't take your advice, they don't even write it down – those are the frustrating things I think” (Psychiatry Consultant 1).

Most of the professionals had strong ideas about how to improve the organisational aspects of the multidisciplinary group meetings. They provide suggestions including better use of time; reducing the number of multidisciplinary group meetings to make better use of professionals’ time; reduce effort to participate by using technology. One example is to merge local groups in order to enhance participation and benefit the facilitation of many small multidisciplinary group meetings.

“I think MDG meetings could be broadened. You know, we're talking about doing that, where you maybe get a couple of local groups together, or now maybe if it's going to be handler-wide we could do something with the other handler group” (General Practitioner 8).

Another participants elaborates on better facilitation processes to overcome time constraints and the fact that the meetings are long by “…chair it [the meeting] a bit more strictly and be, okay, what's your opinion, what's your opinion, what's your opinion, right, let's summarise that and move on” (General Practitioner 2).

Bringing patients to the meetings to enhance the feeling of partnership rather than service delivery was also suggested.

“…I mean, we see ourselves as partners rather than just providers of a service and I think it works both ways, because one of the things that we also argued should be available, is the ability to bring patients who are in-patients to these meetings so that we could discuss with their GPs” (Geriatric Specialist).

Finally technicalities could be overcome by using technology as one participant suggested:

“One of the things that we had suggested earlier on was maybe doing something by Skype, so that you get the benefit of seeing who you're talking to, but also the benefit of not having to travel anywhere, because as soon as you make travelling a requirement, it does limit how many people can go, or the time frame that you have available” (Geriatric Specialist).

## Discussion

The aim of the study was to ascertain the views and experiences of professionals participating in multidisciplinary group meetings within the North West London Integrated Care Pilot context. The perception of integration in this study moves beyond the context of health services used elsewhere [9] to integration of both at the organisational level and at the level of the participating individuals. There was good agreement between and within the author's coding which suggests good reliability of the coding scheme.

The thematic analysis initiated participants’ accounts of benefits of and barriers for participating in the multidisciplinary group meetings. Professionals accounted for benefits in terms of shared learning and integration in three levels, between services, between primary and secondary care and between primary specialists from different practices. On the other hand, participants mentioned that time constraints, multidisciplinary group meetings’ group dynamics and technicalities like quality and timeliness of meetings may restrict their participation. Consequently they offered areas they consider are available for improvement in order to make them active partners rather than just providers of a service.

### Possible explanations in relation to other studies

First, participants indicated that the benefits of multidisciplinary group meetings identified in this study like shared learning should be transparent to individuals and services that are not participating in the multidisciplinary group meetings so that integration is reflected in all levels of health care provision not only within the multidisciplinary group meetings. If this is achieved then shared knowledge should inform clinical practice which currently is lacking in an integrated care context [8–10]. This problem may also reflect the lack of clarity when it comes to defining integrated care in applied settings and the need for standardisation of procedures of implementing the multidisciplinary group meetings. This is not only important for active participants but it is for the benefit of the whole system. It was noted previously that individuals and services may work in an integrated way on an individual patient or case but this may not extend to other individuals, patients or services [13]. Participants also usefully suggest bringing patients to the meetings to enhance partnership and common goal achievement.

Second, the need to enhance quality of communication and collaborative willingness seen elsewhere [22] were also noted in this study. Health professionals’ lack of collaborative working noted in the literature [16, 17] can also inform these findings pointing out the need to structure of multidisciplinary group meeting procedures so that they make their participation easier. Learning was an underlying mechanism describing different perceived benefits and seems to drive the facilitation and participation of professionals in the multidisciplinary group meetings. Participants evaluated the *content*, *organisation* and *implementation* of the multidisciplinary group meetings. Negative evaluations of the content were lack of depth regarding information provision, lack of a sense of accomplishment, retrospective discussion and the lack of structure on organising the multidisciplinary group meetings resulting in variability of quality. Negative evaluations regarding the organisation of multidisciplinary group meetings were the lack of strategy on the cases brought to the meetings with the difficult cases first and then “struggling” to choose cases, short notice to attend the meetings, having multidisciplinary group meetings collapsing, and lack of pointing out the outcomes of the discussion. Negative evaluations regarding the implementation process of the multidisciplinary group meetings were lack of transparency of learning from the participants of the multidisciplinary group meetings to other members of the health care teams and focusing on long-term patient management when primary care providers mostly focus on managing current patients. These findings also reflect the validation elements within the Models of Team Health Care Practice framework [36] like group structure, process and outcomes. The structure of the multidisciplinary group meetings in the North West London Integrated Care Pilot, the diverse background of participants and the infrastructure used can impact the process of how participants interact and relate within the group and the outcomes of the meetings. Valence (extent to which participants support each other) was seen in our previous work with multidisciplinary group meetings [9]. Inter-group communication was also found previously to be affected by obstacles like medical talk [25] and lack of shared goals and objectives [37]. Therefore, emphasis should be given to clarifying and simplifying the processes and outcome procedures of the multidisciplinary group meetings.

Third, professionals in their reflections pointed out the necessity to tackle the barriers of participating in the multidisciplinary group meetings by managing their time and resources. In general, lack of standardised procedures evident elsewhere [16] is also reflected in this study. Participants focused on the need of a sense of partnership rather than provision of a service only. Professionals reported mainly organisational barriers as proposed previously by other studies [24, 38]. Time restrictions were seen as strong barriers among most of the professionals for participating in the multidisciplinary group meetings. Providers raise the concern of limiting the length and frequency of multidisciplinary group meetings, the time commitment prior to participating for preparing and the restriction of devoting their time into seeing their patients especially among primary care providers. Communication and inter-professional understanding is necessary to enable sufficient and effective outcomes not only for the long-term patient care but also for fostering change in individual cases discussed within the multidisciplinary group meetings. In line with a previous evaluation [39] which identified that inter-professional collaboration is not necessarily improved through the integrated care process, this study confirms the importance of identifying how multidisciplinary group meetings are delivered in order to improve health care professionals experience.

Fourth, perceptions of integration in the Integrated Care Pilot seem not universal and clear – therefore the definition of integrated care needs to be re-visited: is it integration of services, knowledge, between sectors, between professionals? At the micro-level integration concerns the individual level while at the meso- and macro-level it concerns the services level [5]. The problem with current definitions of integrated care reflects the fact that even though some take patient care in focus, they don't include all the relevant components of integrated care [40]. Integrating services can help with structuring integration of health care provision along with the collaborative willingness of participants and their learning drive to achieve the integration objectives. Moreover, this study found that integration of care can be achieved through two dimensions of collective action: (a) between organisational settings and (b) between professionals. Using a previous typology of collective action between professionals [41], we propose focusing on enhancing the *relational dimension* of shared goals and vision regarding multidisciplinary group meetings’ implementation and outcomes, by internalising collective action through mutual respect and translation of a sense of belonging, and also through the *organisational dimension* of structuring formalisation [42] of expectations and responsibilities. Also, making use of governance procedures can provide a common direction between the professionals.

### Implications for clinicians and policymakers

The study illustrates the need to re-consider the role and facilitation of multidisciplinary group meetings within integrated care and to evaluate the professionals’ needs and considerations to motivate them not only to physically participate but also to take ownership and partnership at implementing integrated care in the English National Health System. Therefore, the study also strengthens the argument of re-visiting the quality, implementation and organisation of multidisciplinary group meetings in order on the one hand to minimise the barriers for professionals to participate and on the other hand to enhance the positive evaluations of learning and integration. Moreover, in this study several professionals suggested that as they stand, the multidisciplinary group meetings have more impact on them rather than patient care. This study highlights the disconnect between one of the main aim of the multidisciplinary group meetings which is to improve patients’ experience [9, 13] and the finding that actually multidisciplinary group meetings are more focused on health care professionals’ experience and participation. This should be considered in designing future steps for integrated care in National Health System London in order to make effective use of professionals’ time as well as resources. These areas of action should be addressed holistically to improve providers’ experience from participating in the multidisciplinary group meetings in order to positively impact on patients’ health care provision. This can also respond to previous evaluations [11] which suggest that patient experience is not improved by the implementation of current integrated care systems.

The findings also suggest that policymakers should consider how the multidisciplinary group meetings can have more tangible benefits to the patients through improving health professionals’ participating experience. Lack of standardised procedures were previously shown to constitute a barrier to health professionals’ productive participation in multidisciplinary group meetings [16] and in this study participants offered a range of ideas on how to overcome this problem. Effective multidisciplinary work can also help health professionals change their everyday practice [15] and help patients understand the health care process [19]. There is potential that these findings can help to achieve tangible benefits since young health professionals are positive towards multidisciplinary working, learning and collaboration [20].

### Future directions for research

It is important to note that patient empowerment in integrated care should also be addressed to enable patients to participate in shared decision-making [43]. In this study, professionals provided suggestions for improvement focusing on the professional (i.e. better use of time, reduce effort to participate, using technology). Therefore, professionals’ perceptions seem to shift the focus of integrated care away from patient-centred care provision and patient-provider shared decision-making. Future studies should compare the views of different professionals (e.g., general practitioners, specialists, social workers, etc.) within the multidisciplinary group meetings, to identify how we can re-design such groups to work better for variety of professions. They should also compare the views regarding integrated care between professionals and patients.

In terms of differences between health professionals there was no clear indication of different perceptions especially between primary and secondary care professionals as opposed to previous evidence pointing out that non-medically trained professionals find it difficult to participate in the discussions [31]. However, we could not find similar findings in our study and more research is needed to tackle health professionals’ contribution to the multidisciplinary group meetings. In our study, health professionals from secondary care discussed more in depth how multidisciplinary group meetings were organised and facilitated. There was an indication in this study towards the group dynamics within the multidisciplinary group meetings pointing out that the groups can be medically focused which makes participation challenging. However, more research is needed in order to be conclusive about the role of group dynamics in professionals’ participation in the multidisciplinary group meetings.

### Strengths and weaknesses of the study

The study has limitations. Professionals interviewed were participating in the North West London Integrated Care Pilot. Therefore, they may be biased in their conception of integrated care and may reflect a percentage of professionals who have been interested or involved at some level with integrated care. This problem reflects the fact that the study uses qualitative data from a purposive, non-randomised sample. We did not have access to participants’ demographics and therefore our findings may be limited to experiences from certain areas, services, genders and levels of experience. Also, the generalisability of the study's findings is limited because of setting and health care context in a single location in North West London. Therefore, studies with a more diverse patient population and professionals may contribute to improved generalisability. Also, we could not perform any further analyses to investigate different perceptions between specialties as most of the participants were medically trained (general practitioners or secondary care clinicians) with some specialties like nurses and social workers underrepresented.

Despite these limitations this study provides useful insight to the implementation and facilitation of multidisciplinary group meetings within an integrated care context in National Health System England that can be used to improve integration of care for the benefit of both professionals and patients. To our knowledge there is no other study exploring the perceptions and experiences of multidisciplinary working among professionals within the context of integrated care. Even though this study focuses on health professionals’ experiences only, these experiences are important in order to evaluate any type of intervention and in order to refine future multidisciplinary group meetings. Health professionals’ views can also help policymakers to design multidisciplinary group meetings in such a way in order to improve and enhance professionals’ participation.

The complexities of integrated care calls for a continuous effort to understand and improve its implementation [44]. Therefore, there is a need for studies evaluating the use of the tools used for implementing integrated care like the multidisciplinary group meetings. The study findings can be used to address the difficulties of health care professionals who are called to deliver the multidisciplinary group meetings. Furthermore, this study strengthens the argument that in order to improve integrated care, policymakers need to look beyond the general implementation procedures and identify the perceptions of those who are called to deliver the procedures and also how the procedures are implemented. Therefore, by improving the tools which support the implementation (like multidisciplinary group meetings) as raised by the interviewees, the care process and the experience for both patients and providers can be also improved and vice versa.

## Conclusion

This study's findings provide insights in improving current implementation of integrated care and the design of future interventions within the English National Health System. Multidisciplinary group meetings are beneficial, but it is not clear that they can be beneficial for the patient health care. They seem beneficial for the professional mostly. Multidisciplinary group meetings have the potential to contribute to integration of care but need to build on the elements that work (learning processes, sharing information) and clarify the direction of learning (towards patient care rather than the professional only) overcoming the barriers for participation (time restrictions, group dynamics, technicalities around the facilitation processes). The learning process can be used to benefit patient health care provision. It can also be that if professionals are partnered together with common goals and objectives rather than feeling as providers of a service can benefit the multidisciplinary group meeting implementation and integration of care for the benefit of the professional, the patients and the health care system.

## Appendix

### Interview Schedule and Topic Guide of North West London Integrated Care Pilot: Qualitative evaluation of integration and patient and practitioner experience.

#### Draft topic guide for Health and Social Care Practitioners

##### Knowledge and attitude

What do you know about the Integrated Care Pilot?

How did you learn about the Integrated Care Pilot? (*Probe: sources, types of information and timing*)

What do you think is the pilot trying to achieve? (*Probe: is there a clear vision?*)

How do you know these are the aims? (*Probe: any meetings/presentations run to spread the message/engage with staff?*)

Do you believe that, if delivered effectively, the integrated care partnerships could deliver better care for patients? How? How would you know if it had delivered better care? What would that look like?

What are your thoughts about the Integrated Care Pilot (*can we probe thoughts about the overall aims/vision AND thoughts about how the pilot is being operationalised? Why did they sign up in a first place? We need to try and understand if they feel engaged and why*)

What are your thoughts about the model of care within the pilot? How is it different to “usual care”?

##### Practice (in each case probe for examples)

What changes has the pilot involved for you?

Was any training provided?

By whom, when, adequacy, relevance

How has the pilot affected the way you do your job? (*Probe: use different information or the same information differently; communicate with different people or in a different way; use of shared protocols*)

How has the pilot affected the way you work with patients? (*Probe: in terms of your relationship with them and/or in terms of what information you share with them or the types of discussions you have with them*)

Has the pilot affected the care provided? (*Probe: do the patients see the same people in the same settings with the same choices and the same information as before?*)

What differences would patients notice?

##### Influences on integrated care, process and outcome

Given the aims of the pilot as you set them out (above), do you think integrated care is being delivered to patients? (*Probe: if what way – examples of what's different now to before?*)

What has influenced the way integrated care has been provided (or what factors are determining whether the aims are being achieved or not)? (*Probe: system, patients, funding, contextual factors – e.g. clinical protocols, financial incentives, other administrative support, IT tool, in what way, ask for examples*).

How has the pilot affected or interacted with any other changes, restructuring or service activities, National Health System reforms?

To what extent does integrated care feel integrated in the system? (*Probe: changes in infrastructure, part of normal business, relationship to other systems.*)

Have you experienced any barriers or obstacles in your attempts to offer integrated care?

What has influenced the outcomes of care in the pilot?

How could integrated care be improved?

Can you envisage this being routine practice in the future? What needs to happen to make it routine?

Would you recommend integrated care as it's practiced here to another service.
